# Tryptophan recovery index as a new biomarker for fitness

**DOI:** 10.17179/excli2022-4889

**Published:** 2022-06-24

**Authors:** Alexander Pichler, Andreas Meinitzer, Dietmar Enko, Peter Schober, Georg Singer, Christoph Castellani, Markus Herrmann, Sandra J. Holasek, Holger Till, Jana Maria Windhaber

**Affiliations:** 1 Division of General Anesthesiology, Emergency - and Intensive Care Medicine, Medical University of Graz, Graz, Austria; 2 Clinical Institute of Medical and Chemical Laboratory Diagnostics, Medical University of Graz, Graz, Austria; 3 Department of Pediatric and Adolescent Surgery, Medical University of Graz, Graz, Austria; 4 Division of Immunology and Pathophysiology, Otto Loewi Research Center, Medical University of Graz, Graz, Austria

**Keywords:** tryptophan, kynurenine, kynurenic acid, kynurenine pathway, exercise

## Abstract

The maximal oxygen uptake (VO_2_max) and maximal power output (P_max_) are commonly used parameters to evaluate the endurance fitness status. A connection between exercise and the kynurenine pathway (KP), which describes the metabolism of unused tryptophan, has already been reported. However, a potential association of the KP with endurance fitness levels remains unknown. In this study, adolescent competitive athletes performed an exhaustive incremental exercise test. Blood samples were taken before, directly after, and 30 minutes after the end of exercise. Tryptophan (Trp), kynurenine (Kyn) and kynurenic acid (KA) serum levels were determined by high-performance liquid chromatography (HPLC). Forty-four male and 27 female athletes (median age: 16 years) were recruited. During exhaustive exercise tests, Trp initially declined and then increased 30 minutes after discontinuing exercise. Similar findings were observed for Kyn, whereas KA levels behaved inversely. After incremental exhaustive exercise the relative increase of Trp concentrations, termed the tryptophan-recovery-index (TRI), showed a highly significant positive correlation with VO_2_max and P_max _(r=0.468 and 0.491, p-values <0.001). There was a significant gender-difference with higher levels of all metabolites at all measured time points in male participants. In the present study, a highly significant correlation was found between the TRI and the maximal oxygen uptake in well-trained athletes. The implementation of TRI can therefore be suggested as a biomarker for physical fitness.

## Introduction

The evaluation of fitness is a much-debated topic not only amongst sports medicine specialists, but also in popular science. While the ideal fitness parameter depends on the kind of sport, the maximal oxygen uptake (VO_2_max) and the maximal power output (P_max_) during an exhaustive incremental exercise test are commonly used markers. Beside these the aerobic and anaerobic threshold are also frequently applied.

Recently, different authors investigated a possible connection between exercise and the kynurenine pathway (KP) (Martin et al., 2020[18]). This pathway describes the metabolism of approximately 90-99 % of the unused essential amino acid tryptophan (Trp) (Palego et al., 2016[23]; Richard et al., 2009[25]; Stone and Darlington, 2002[27]). The first enzymes of the KP are the tryptophan-2,3-dioxygenase (TDO) and different subtypes of the indoleamine-2,3-dioxygenase (IDO) (Chen and Guillemin, 2009[7]; Ball et al., 2007[5]), which metabolize Trp to kynurenine (Kyn). Since the main stimulator of IDO is interferon-γ (IFN-γ), the expression and activity of IDO are strongly related to inflammatory processes (Chen and Guillemin, 2009[7]; Grant et al., 2000[11]). Consequently, changes in the KP have been described in a variety of diseases (Nagy et al., 2017[22]), also including psychiatric disorders, such as depression (Chen and Guillemin, 2009[7]; Song et al. 2017[26]). While exercise is thought to be beneficial in individuals with mental disorders, it was supposed that the mood enhancing effects of exercise could be caused by changes of the KP (Allison et al., 2019[3]; Agudelo et al., 2019[2]).

Results of previously published studies examining exercise related alterations of the KP are inconsistent. Data have shown a decrease of Trp while Kyn mostly remained unchanged. Currently, the exercise induced kinetics of KA are poorly investigated (Metcalfe et al., 2018[20]). It is not fully understood yet, why the KP changes after exercise and how exercise can influence the KP. While many authors described a higher activity of KP-enzymes (Strasser et al., 2016[28][29]; Ito et al., 2003[14]; Koliamitra et al., 2019[16]) a shift of KP-metabolites into other body compartments could be another explanation for the metabolic kinetics (Areces et al., 2015[4]). In trained athletes, a permanent adaptation of the KP can be hypothesized. As a consequence, it may be possible to deduce the fitness level by investigating alterations of the KP. This is of great interest, considering that there is no biochemical parameter available, which can estimate somebody's fitness level. 

Therefore, the aim of the present study was to investigate possible associations between the kinetics of Trp metabolites and fitness parameters. We studied adolescent trained athletes during incremental exhaustive exercise testing for changes in the KP. The main focus of this study was on the recovery efficiency. 

## Materials and Methods

### Study population and exercise testing

A total of 71 athletes (44 males, 27 females) were included in this study after written informed consent of the patient or the legal guardian. The inclusion criteria were athletes aged between 14 and 18 years, visiting the outpatient clinic for a voluntary performance review. Each study participant underwent a clinical examination for the suitability of a physical load (12-lead ECG), determination of the resting heart rate (Assy Cam 14®, GE Healthcare, Chicago, Illinois, USA), resting blood pressure (oscillatory, Boso Medicus®, BOSCH+SOHN, Jungingen, Germany), physical examination, an estimation of the total body fat (Caliper, John Bull, British Indicators Ltd., St Albans, UK) (Jackson and Pollock, 1978[15]), and a history of the accomplished training. Athletes were excluded from the study in case of incomplete study parameters. 

An exhaustive incremental exercise protocol was performed on a bicycle ergometer (Excalibur Sport®, Lode, Groningen, Netherlands). During the test, we recorded a 12-lead ECG and a continuous spirometry (both Jaeger Oxycon Pro™, Hoechberg, Germany), and a pulse oximetry (BCI® Autocorr® Pulse Oximeter, Minneapolis, USA). Blood was drawn from an indwelling venous cannula before (t_1_), immediately after the exhaustive incremental exercise test (t_2_), and after a 30 minutes recovery period (t_3_). 

Ethical approval was provided by the local Ethical Committee 27-406 ex 14/15. The study was performed according to the declaration of Helsinki. Informed written consent was provided by all participants. 

### Analysis of KP key metabolites

Blood samples were drawn with 2.8 mL standard serum tubes (*Vacuette®*, *Greiner Bio-One*, Kremsmünster, Austria) and were allowed to clot for 30 minutes. After centrifugation at 4000 x g for 10 minutes, 1 mL serum portions of the supernatant were harvested and stored at -80 °C until further evaluation.

Trp, Kyn and KA were measured by high-performance liquid chromatography (HPLC) coupled with a simultaneous ultraviolet and fluorometric detection system (Hervé et al., 1996[12]; Xiao et al., 2008[30]). All evaluations were performed according to the published guidelines (FDA, 2018[10]). Within-day coefficients of variation (CVs) at different concentrations were in the range between 1.7 % to 4.3 % for Kyn, 0.7 % to 2.9 % for Trp and 2.6 % to 4.5 % for KA. The between-day CVs were 2.0 % to 5.4 %, 6.3 % to 9.3 %, and 8.4 % to 11.6 %, respectively. 

### Statistical analysis 

Non-normally distributed data were displayed as medians and interquartile ranges (IQR). Correlation analyses between parameters were performed using a Spearman's rho (ρ) test. For comparison of independent groups (gender differences) a Mann-Whitney-U-test was applied. Differences between dependent variables (KP kinetics) were examined with a Friedmann-test. In case of significant results a pairwise comparison with a Wilcoxon-test followed by the Bonferroni-Holm correction for multiple testing was performed. Associations between parameters were calculated with simple linear regression model. P-values <0.05 were regarded as statistically significant. Statistical analysis was performed with SPSS® Statistics 22 (*IBM®,* Armonk, USA).

Tryptophan recovery index (TRI), kynurenine recovery index (KRI) and kynurenic acid recovery index (KARI) were calculated by the following formulae:


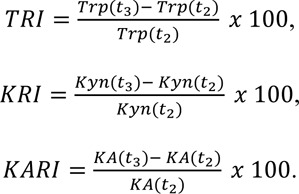


## Results

### Study population characteristics

A total of 71 athletes (44 males, 27 females) with a median age of 16 (range: 14-18) years were examined. Detailed data for the participants are given in Table 1(Tab. 1). Median concentrations of Trp, Kyn and KA at t_1_ were significantly higher in men compared to women (Trp: 70.3 vs. 60.3 µmol/L, p=0.004; Kyn: 3.06 vs. 2.66 µmol/L, p<0.001; KA: 47.1 vs. 39.9 nmol/L, p=0.005). 

### Exercise induced KP kinetics

**Figure 1:**
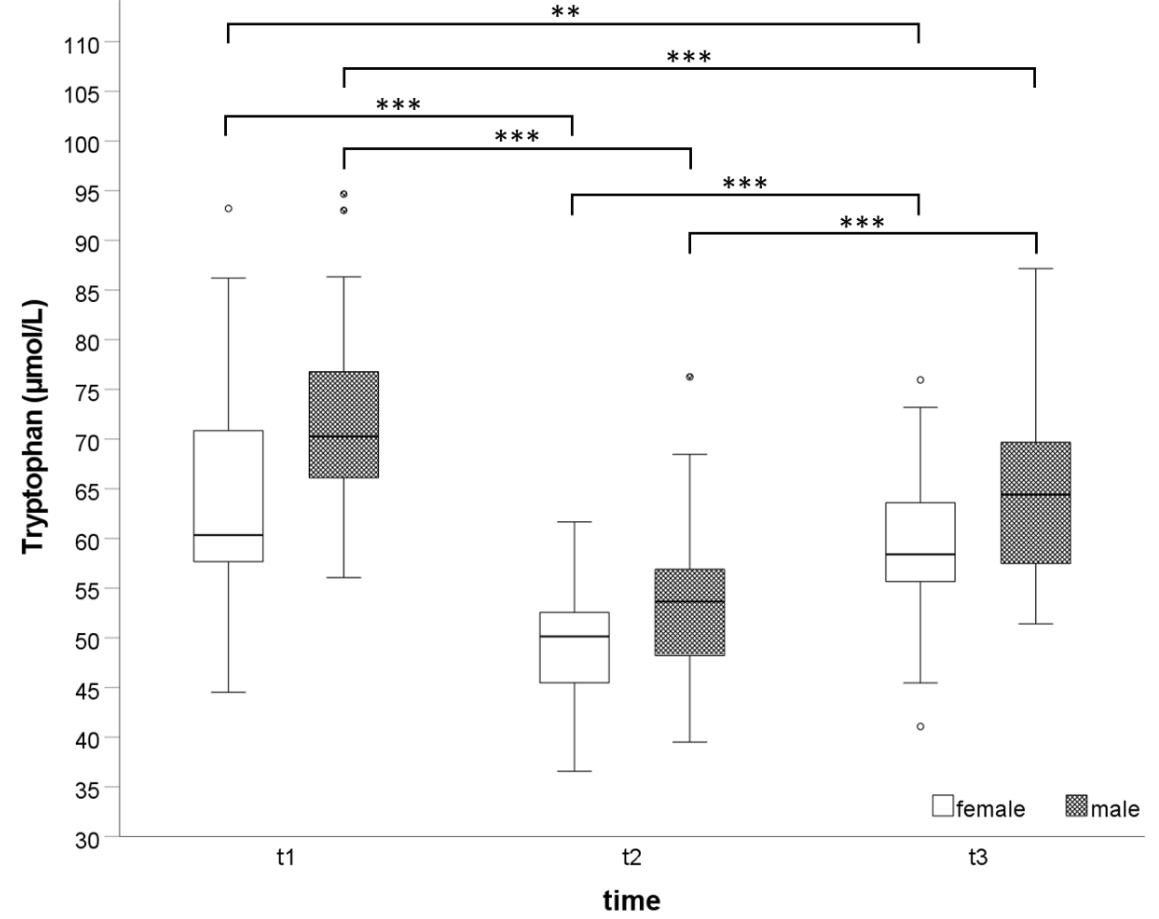
Exercise induced kinetic of Trp blood concentrations before (t1), immediately after an exhaustive incremental exercise test (t2), and after 30 minutes of recovery (t3). **p=0.003; ***p<0.001

Independently from sex, Trp and Kyn concentrations significantly decreased after exhaustive effort (t_2_ vs. t_1_), while KA significantly increased (all p-values ≤0.001). At t_3_ Trp and Kyn concentrations increased (all p-values <0.001) while KA (p<0.001) decreased significantly compared to t_2_. At t_3_, Trp was still significantly lower (p<0.001 in men and p=0.003 in women) than at t_1_. However, there was no significant change between Kyn concentrations between these points of time. Only in men, KA concentrations were significantly increased at t_3_ compared to t_1_ (p < 0.001). The kinetic of Trp blood concentrations is demonstrated in Figure 1(Fig. 1).

### Correlations between kinetics of KP metabolites with fitness parameters

**Figure 2:**
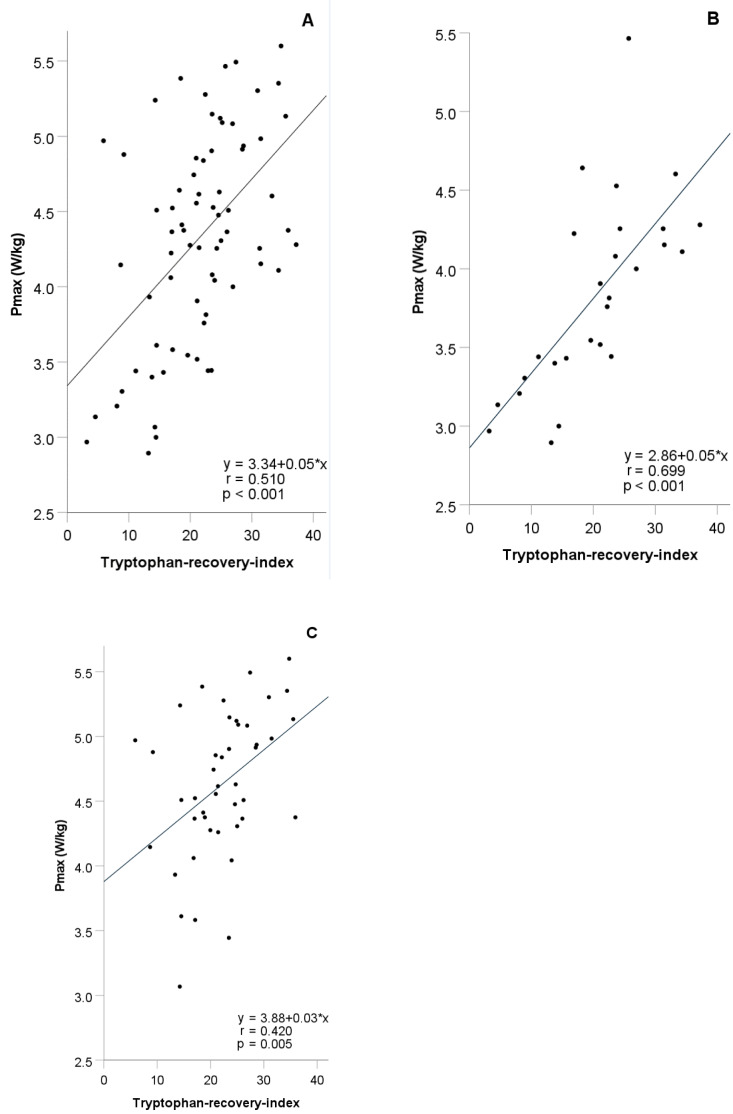
Simple linear regression analysis between tryptophan-recovery-inex and P_max_ in all (N=71) (A), female (N=27) (B), and male athletes (N=44) (C)

After incremental exhaustive exercise the relative increase of Trp concentrations (TRI) showed a highly significant positive correlation with VO_2_max and P_max _(r=0.468 and 0.491, p-values <0.001). The correlations of VO_2_max and P_max _were stronger in female (r=0.713 and 0.794, p-values <0.001) than in male athletes (r=0.429 and r=0.452, p=0.004 and p=0.002). A significant positive correlation between the relative increase of Kyn concentrations (KRI) and fitness parameters (VO_2_max and P_max_) (r=0.433 and 0.581, p= 0.024 and p=0.001) was observed in females, only. No significant correlation was found between the relative decrease of KA (KARI) and fitness parameters. Detailed results of the TRI are shown in Table 2(Tab. 2) and Figure 2(Fig. 2). The anonymized raw data of the present study are provided in Supplementary data (Table 1). 

## Discussion

In this study, we found a strong correlation between the increase of Trp concentrations after exercise and fitness parameters in adolescent athletes. To the best of our knowledge, this is the first study describing the ratio of Trp concentrations immediately and 30 minutes after exercise in correlation to the fitness parameters VO_2_max and P_max_. This ratio, called TRI, describes the recovery efficiency of Trp. 

We observed a decrease of Trp after exercise, which is in line with previous studies in animals and humans (Metcalfe et al., 2018[20]; Strasser et al., 2016[28][29]; Areces et al., 2015[4]; Mudry et al., 2016[21]; Lewis et al., 2010[17]; Bansi et al., 2018[6]; Melancon et al., 2014[19]). In rats, Ito et al. observed a mean decrease of 43.5 % of blood Trp concentrations after treadmill exercise with exhaustion (Ito et al., 2003[14]). Mudry et al. found slightly decreased Trp levels in diabetic patients and healthy controls after 30 min training compared to baseline concentrations (Mudry et al., 2016[21]). Also in athletes Trp declined after exhaustive exercise (Strasser et al., 2016[28][29]), even during ultramarathon running (Yamada et al., 2016[31]). In our study, we observed a decline of Kyn after exercise. In contrast to Trp, contradicting results have been published regarding Kyn alterations after exercise. Data from Yamada et al. (2016[31]) are consistent with our findings. They found a decrease of Kyn after 35 km ultramarathon running. However, other authors describe an increase of Kyn after exercise (Strasser et al., 2016[28][29]; Mudry et al., 2016[21]; Ito et al., 1999[13]). Contrary to Trp and Kyn, we found a distinct increase (approximately 40 %) of KA after exercise. Little data are available about alterations of KA after exercise. Our findings confirm one previously published study (Mudry et al., 2016[21]). 

Nevertheless, the explanation for alterations of Trp and KP metabolites during and after exercise is an emerging topic of current discussions. Previous studies reported a shift of amino acids due to exercise from the vascular compartment into skeletal muscle (Strasser et al., 2016[29]; Areces et al., 2015[4]). This shift may be facilitated by an enhanced expression of the large amino acid transporter 1 (LAT1), which carries Trp into skeletal muscle (Martin et al., 2020[18]; Pillon et al., 2020[24]). A higher physiological energy consumption in skeletal muscle could be one possible explanation for the observed Trp kinetics for energy production (Agudelo et al., 2019[2]). Moreover, during exercise, the PPAR-γ coactivator-1α1 (PGC-1α1) enhances the expression of kynurenine-amino-transferases (KATs) in skeletal muscle elevating the production of KA (Agudelo et al., 2019[2]; Agudelo et al., 2014[1]). Subsequently, more KA is shifted from skeletal muscle into the circulation (Martin et al., 2020[18]). This theory is in line with our data showing a Kyn decrease and KA elevation after exercise. Allison et al. showed that a 12-week exercise program increases the expression of skeletal muscle transcription factors PGC-1α, PPARα and PPARδ, but they did not see an effect on the kynurenine metabolism (Allison et al., 2019[3]).

We found a strong association between the TRI and the conventional fitness parameters. In comparison Strasser et al. described a positive correlation between the maximal oxygen consumption (VO_2_max) and values of Trp (Strasser et al., 2016[29]). However, they did neither assess the association between the rebound of Trp after a recovery period nor the fitness status. 

It is well known that physical exercise activates the coactivator PGC-1α1, a master coactivator of cellular adaptive processes, which improves fuel supply, uptake and utilization (Correia et al., 2015[8]), but also the expression of KATs (Agudelo et al., 2014[1]). The increased activity of the KATs might lead to higher blood glutamate concentrations, which are important for energy utilization. Moreover, the PGC-1α1 regulated pathway in the trained muscle is considered to use Kyn as a supporter for aspartate biosynthesis and mitochondrial function (Agudelo et al., 2019[2]). It might be possible that in trained athletes this pathway is gained. After exercise the no longer needed Trp and Kyn is shifted back faster into the vascular compartment in better trained athletes. Therefore, the TRI is higher in these individuals. 

Male athletes had a higher VO_2_max compared to female athletes, which correspond with the higher baseline values of the KP. This gender difference was already discussed in prior studies (Strasser et al., 2016[29]). It was suggested that estrogen and progesterone activate the KP downstream (de Bie et al., 2016[9]). However, we found significantly higher Trp, Kyn and KA in men, which partly is in line with a previous report describing higher Kyn blood concentrations in men, but no differences in other KP metabolites (Strasser et al., 2016[29]). Another study of the same study group reported lower Kyn levels in women (Strasser et al., 2016[28]). In contrast to conventional fitness parameters, the TRI might give information about the individual fitness status independently from sex. 

Further studies are required to optimize the predictive value of the TRI including duration and degree of the necessary exercise, duration of observation and recovery. If it is possible to determine the TRI in a simple way, it could be a valuable parameter to evaluate the fitness status.

Some limitations of this study should be mentioned. The last meal before blood taking was not standardized due to comfort for our athletes. This circumstance could have an influence on the baseline values of Trp metabolism. Moreover, inflammatory markers, such as interleukin-6 (IL-6) or cortisol were not assessed. 

## Conclusions

In the present study, a highly significant correlation could be found between the TRI and the maximal oxygen uptake in well-trained athletes. The authors of this study suggest to use the TRI as a biomarker for physical fitness. 

## Conflict of interest

The authors declare that there is no conflict of interest.

## Supplementary Material

Supplementary data

## Figures and Tables

**Table 1:**
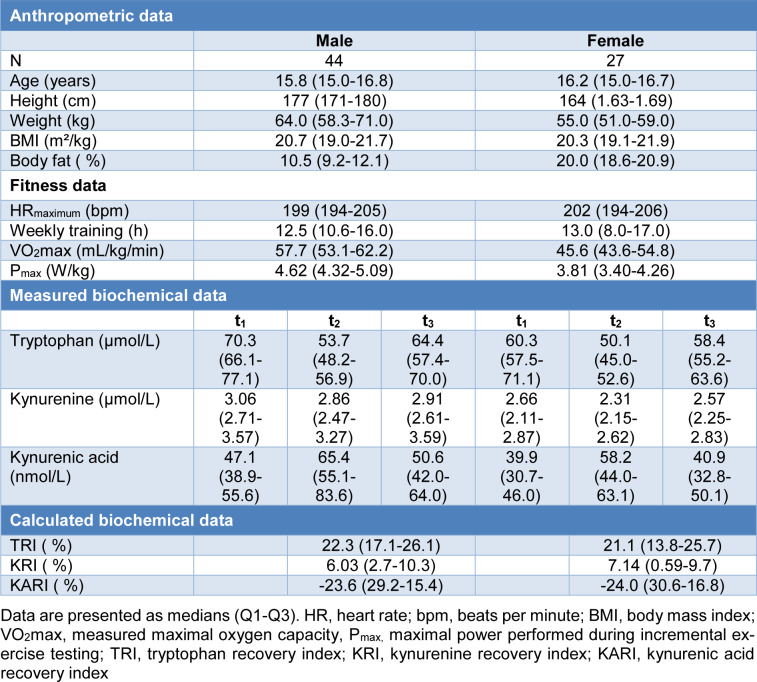
Anthropometric and biochemical characteristics of participants

**Table 2:**
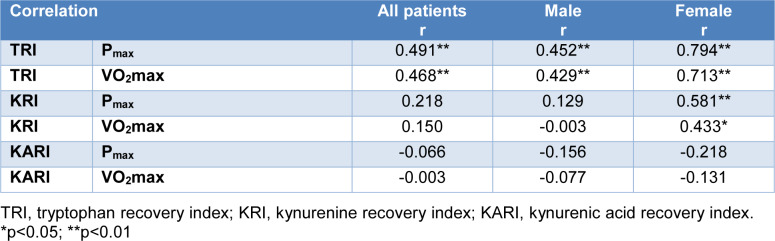
Simple linear regression of TRI, KRI and KARI with fitness parameters
